# COVID-19 trends, public restrictions policies and vaccination status by economic ranking of countries: a longitudinal study from 110 countries

**DOI:** 10.1186/s13690-022-00936-w

**Published:** 2022-08-24

**Authors:** Myung-Bae Park, Chhabi Lal Ranabhat

**Affiliations:** 1 Department of Gerontology Health and Welfare, Pai Chai University, Daejeon, Republic of Korea; 2 Department of Health Promotion and Administration, Eastern Kentucky University, Richmond, KY USA; 3 Global Center for Research and Development, Kathmandu, Nepal

**Keywords:** COVID-19, SARS-CoV-2, Mortality, Reproductive ratio, Stringency index, Vaccine

## Abstract

**Background:**

The coronavirus disease 2019 (COVID-19) pandemic has transitioned to a third phase and many variants have been originated. There has been millions of lives loss as well as billions in economic loss. The morbidity and mortality for COVID-19 varies by country. There were different preventive approaches and public restrictions policies have been applied to control the COVID-19 impacts and usually measured by Stringency Index. This study aimed to explore the COVID-19 trend, public restriction policies and vaccination status with economic ranking of countries.

**Methods:**

We received open access data from Our World in Data. Data from 210 countries were available. Countries (*n* = 110) data related to testing, which is a key variable in the present study, were included for the analysis and remaining 100 countries were excluded due to incomplete data. The analysis period was set between January 22, 2020 (when COVID-19 was first officially reported) and December 28, 2021. All analyses were stratified by year and the World Bank income group. To analyze the associations among the major variables, we used a longitudinal fixed-effects model.

**Results:**

Out of the 110 countries included in our analysis, there were 9 (8.18%), 25 (22.72%), 31 (28.18%), and 45 (40.90%) countries from low income countries (LIC), low and middle income countries (LMIC), upper middle income countries (UMIC) and high income countries (HIC) respectively. New case per million was similar in LMIC, UMIC and HIC but lower in LIC. The number of new COVID-19 test were reduced in HIC and LMIC but similar in UMIC and LIC. Stringency Index was negligible in LIC and similar in LMIC, UMIC and HIC. New positivity rate increased in LMIC and UMIC. The daily incidence rate was positively correlated with the daily mortality rate in both 2020 and 2021. In 2020, Stringency Index was positive in LIC and HIC but a negative association in LMIC and in 2021 there was a positive association between UMIC and HIC. Vaccination coverage did not appear to change with mortality in 2021.

**Conclusion:**

New COVID-19 cases, tests, vaccinations, positivity rates, and Stringency indices were low in LIC and highest in UMIC. Our findings suggest that the available resources of COVID-19 pandemic would be allocated by need of countries; LIC and UMIC.

## Background

The World Health Organization (WHO) declared the outbreak of coronavirus disease 2019 (COVID-19) a global pandemic on March 11, 2020. The mortality rate associated with COVID-19 is dependent on the number of confirmed cases [1]. As of May 2022, the cumulative number of confirmed cases and deaths was more than 5.3 billion and 6.3 million, respectively [2]. Meanwhile, the countries with the highest mortality rates for the same period were San Marino, Belgium, Slovenia, and the United Kingdom [3]. The case fatality ratio varies among countries; however, it has been estimated as 3–9% in the early stages of the pandemic [4, 5]; as of January 2021, it is estimated at 2% [1]. Concurrently, mortality rates among patients hospitalized for COVID-19 infection have been estimated at 18.9% [6]. It shows the multiple disparities of COVID-19 trends like region, economic ranking, place of mobility etc.

COVID-19 testing helps identify and treat infected patients as well as those that may infect others, thus contributing to the prevention of the disease spread [7]. In this context, a real-time reverse transcription-polymerase chain reaction is the preferred testing method [8]. Although the number of confirmed cases and the corresponding restrictions vary among countries, pathogenicity, virulence, controlling effort from government and individual effort protecting from disease [9] [10, 11]. SARS-CoV-2 spreads through respiratory aspirates, droplets, and contact, and has higher transmissibility than do both SARS-CoV and MERS-CoV [12, 13], with an incubation period of 5–15 days [14]. Individuals with asymptomatic infection may infect others via pre-symptomatic or asymptomatic transmission, resulting in global governments imposing restrictions on their citizens, including the use of facilities and travel, broadly known as lockdowns, to curtail any sharp increases in the number of confirmed cases [15]. COVID-19 is more contagious than many other infectious diseases because the variants have been developed rapidly, there are no clear evidence that available vaccines are effective to all variants and severely impacted global economy.

Previous studies have mostly focused on the distribution and determinants of the COVID-19 pandemic by different dimensions such as social determinants of mortality [16, 17], disparities [18], social and mental health [18], food security status [19], and labor markets [20]. The major aspects of disparities were testing of cases, access with testing kids, supply of protective equipment like mask, sanitizer [21] and availability of COVID-19 vaccine [22]. Obviously, countries in power more focused to protect themselves other than globally control of COVID-19 pandemic. So, countries with a poor economy were more seriously affected in different aspects by this pandemic than rich countries. In this context, there is a need to analyze by economic rank of country because it could help resource allocation to prevent pandemic, necessary supply of vaccines and other commodities based on need than demands and smart approaches of daily activities other than hard restrictions like lockdowns and restrictions of basic lives. Likewise, it is necessary to see the trends of common preventive strategies like school closures, workplace closures, and travel bans. In composite index, it is defined as stringency index [23]. Policies and strategies taken to mitigate the COVID-19, similar disease and possible future pandemic depends on available resource and income status of countries [24]. To reduce peak healthcare demand while protecting those most at risk of severe disease from infection, and reverse epidemic growth, reducing case numbers to low levels and maintaining that situation indefinitely public restrictions policies are in practice [25]. Common policies included school closures, travel restrictions, bans on public gatherings, stay-at-home orders, closure of public transportation, emergency investments in the healthcare system, new forms of social welfare provision, contact tracing, and investment in COVID-19 vaccines [26].

Right now, the COVID-19 pandemic seems to be controlled but there are new cases in more than 90 countries, about half a million new cases each day and about one thousand daily mortality [27]. Likewise, there is a high chance originating a new variants and nobody predicts its severity and virulence. According to *Nature*, there are two variant of Omicron, namely BA.4 and BA.5 ongoing rise, as well as the diversity of mutations carried by them [28]. Canadian scientist recently reported its first cases of the Omicron subvariant XE, one of several hybrid variants of SARS-CoV-2 that have emerged since the beginning of this year [29]. Globally, there are a cautious opening of public places for fun, and the economy has not revived at a previous pace. There is a great impact of COVID-19 on survivors, missing families, businesses and countries with poor economic conditions [30, 31]. There is a need of study with convincing source of data which could be global coverage, reliable, and evidence for policy implications. Similar previous studies were primarily cross-sectional, restricted to a handful of countries, or based on univariate analyses. Accordingly, we aimed to investigate the trend of disease and investigation outcomes like daily mortality, incidence, testing, positivity, and the strictness of restrictions; presented with stringency index and vaccination status of whole COVID-19 pandemic with GNI per capita. The stringency index is a composite measure based on nine response indicators to a value from 0 to 100 (100 = strictest) [32]. Those indicators are, school and workplace closure, cancellation of public events and gathering, stay home in restriction, faces covering, public information campaigns, international and domestic travel, testing and contact tracing, vaccination policy, income support and debt relief and google mobility trend [33]. In COVID-19 pandemic, most of the countries applied public restriction however those restrictions have pros and cons and such restrictions may need to apply in similar situation in future. So, this study aims to explore the COVID-19 trend, public restriction policies and vaccination status by economic ranking of countries.

## Methods

For the present study, we used data from Our World in Data, an organization that collects statistics from around the world for educational and research purposes, including global data on COVID-19, provided by the Data Repository by Johns Hopkins University. The Johns Hopkins Coronavirus Resource Center (CRC) is a continuously updated source of COVID-19 data and expert guidance. According to the university, the dashboard uses data from government bodies including the U.S. Centers for Disease Control and Prevention (CDC), the European Center for Disease Prevention and Control (ECDC), and the National Health Commission of China, and other organizations like the World Health Organization (WHO), local media reports, and the DXY, one of the world’s largest online communities for physicians, health care professionals, pharmacies, and health facilities [34]. So, it is considered more reliable. At the time of extraction, data from 210 countries were available but 100 countries without data related to testing, which is a key variable in the present study, were excluded. Consequently, data from 110 countries were used, and the analysis period was set between January 22, 2020 (when COVID-19 was first officially reported) and December 28, 2021.

### Variables

In this study, the daily mortality rate was defined as the daily number of new deaths per one million people. Such data was imported from Johns Hopkins University & Medicine, Coronavirus Resource Center, Maryland [35]. The daily incidence rate was calculated as the number of new confirmed cases per one million people, and the daily testing rate was calculated per 1000 people. In addition, the positivity rate was calculated as the number of confirmed cases per test using a 7-day rolling average. The stringency index reflects the response measures implemented by governments to prevent the spread of infection. This index is based on nine measures, school closures, workplace closures, cancellation of public events, restrictions on public gatherings, closures of public transport, stay-at-home requirements, public information campaigns, restrictions on internal movements, and international travel controls. Its value ranges from 0 to 100 points with higher scores indicating more stringent controls by the government [36]. Vaccine coverage is the number of people vaccinated at least per one million people. All variables in the present study were panel data; time-dependent variables were reported in units per day. Finally, there was no multicollinearity among the independent variables (Table 3 in Appendix).

### Sub-group analysis

All analyses were stratified by i) 2020 and 2021 year and, ii) the World Bank income group. As of October 2021, according to the World Bank, countries could be stratified into low-income countries (LIC; < 1045 USD/capita/year), lower-middle income countries (LMIC, 1046–4095 USD/capita/year), upper-middle income countries (UMIC; 4096–12,695 USD/capita/year), and high-income (HIC; > 12,695 USD/capita/year) countries [37]. By proportion, there are 28 (13.08%) countries in LIC, 54 (25.23%) countries in LMIC, 54 (25.23%) countries in UMIC and 81 (37.85%) countries in HIC out of total 214 countries. In addition, the first vaccine against COVID-19 was launched in the UK on 8 December. In that moment, few countries have started vaccinations because most of these variables are missing in 2020, and we did not include vaccine coverage as the independent variable in the 2020 model.

### Statistics

We performed descriptive analyses on all variables. To analyze the associations among the major variables, we used a longitudinal fixed-effects model (FEM). Although panel data at a regional or national level are not population-extracted data, the FEM is generally more appropriate than the random-effect model. we used the FEM because FEM explored the relationship between predictor and outcome variables within an entity (country, person, company, etc.) [38]. Thereby allowing control of the effects of confounders that could influence the dependent and independent variables, particularly factors that remain constant over time and those that are difficult to observe and measure. We evaluated the model suitability of FEM, which is a longitudinal cross-sectional analysis, not ordinary least square (OLS) [39]. In addition, both the F test and Hausman test results showed Prof > F = 0.0000 and Prob > Chi^2^ = 0.0000; therefore, we confirmed that FEM is a more effective model than the pooled ordinary least squares or random-effect model.

In the present study, the daily mortality rate was set as the dependent variable, and its associations with other major variables were analyzed using the following formula:

$$2 020\ year\ model; Daily\ mortality\ rate\ \left( lnNew\ deaths\ per\ million\right)=\alpha i+\beta 1\cdotp lnNew\ cases\ per\ {million}_{it}+\beta 2\cdotp lnNew\ tests\ per\ {thousand}_{it}+\beta 3\cdotp lnPositivity\ {rate}_{it}+\beta 4\cdotp {lnStringency\ i ndex}_{it}+ Eit$$

$$2 021\ year\ model; Daily\ mortality\ rate\ \left( lnNew\ deaths\ per\ million\right)=\alpha i+\beta 1\cdotp lnNew\ cases\ per\ {million}_{it}+\beta 2\cdotp lnNew\ tests\ per\ {thousand}_{it}+\beta 3\cdotp lnPositivity\ {rate}_{it}+\beta 4\cdotp {lnStringency\ i ndex}_{it}+\beta 5\cdotp lnVaccinated\ people\ per\ {hundred}_{it}+ Eit$$

where i = countries, t = time point (day).

Moreover, independent variables may be subject to a time-lag effect or be independently associated with the dependent variable. The incubation period of SARS-CoV-2 varies between measurement methods and countries [40, 41] with the reported median of 1 week; however, other estimates have also been proposed, including the average of > 2 weeks [14]. To account for this variability, we performed additional analyses with time lags of 15 days.

Lastly, the linear regression line is not necessarily a straight line. Depending on the number of deaths, the strength of the association among each independent variable may vary. Therefore, we created prediction plots using quadratic fixed-effects regression to identify the associations between daily mortality rates and each independent variable. This approach allowed for a more intuitive understanding of the association between two variables. Except for descriptive analyses, all variables included in the models were ln-transformed.

## Results

In our analysis, there were 110 countries and out of those, 9 (8.18%), 25 (22.72%), 31 (28.18%), and 45 (40.90%) countries included from LIC, LMIC, UMIC and HIC respectively.

In 2020, the daily mortality rate was the highest in HIC (1.65 ± 4.78), followed by UMIC (1.59 ± 3.18), LMIC (0.44 ± 2.10), and LIC (0.06 ± 0.20); however, in 2021, the daily mortality rate in UMIC (3.25 ± 6.03) was the highest. In 2020, the daily confirmed incidence rate was the highest in HIC (89.40 ± 216.05), followed by UMIC (61.00 ± 127.45), LMIC (18.01 ± 47.00), and LIC (2.31 ± 4.81). In 2021, daily confirmed incidence rate was the highest in HIC (233.50 ± 483.50), followed by UMIC, LMIC, and LIC. Pertaining to the number of daily tests in 2020, HIC (1.99 ± 2.73) had the highest number, followed by UMIC (0.53 ± 0.72), LMIC (0.29 ± 0.63), and LIC (0.06 ± 0.08). In 2021, HIC (7.45 ± 12.48) had the highest number, followed by UMIC, LMIC, and LIC. Pertaining to the positivity rate in 2020, UMIC had the highest (0.13 ± 0.13), followed by LMIC (0.09 ± 0.09), LIC (0.06 ± 0.08), and HIC (0.06 ± 0.08). In 2021, UMIC (0.12 ± 0.09) had the highest positivity rate, followed by LMIC, LIC, and HIC. In case of the stringency index in 2020, UMIC (65.61 ± 21.50) had the highest, followed by LMIC (60.91 ± 23.17), LIC (56.56 ± 22.49), and HIC (54.93 ± 20.99). In 2021, UMIC (59.3408 ± 15.97) had the highest stringency index, followed by HIC (53.92 ± 16.26), LMIC (50.66 ± 19.66), and LIC (39.75 ± 17.49). Finally, for 2021 the vaccinated people per hundred was the highest in HIC (3.42 ± 16.26), followed by UMIC (2.87 ± 1.18), LMIC (2.39 ± 1.28), and LIC (1.17 ± 0.82) (Table 1). There were not data of vaccination in 2020.

**Table 1:** Summary data for 110 countries since the start of the COVID-19 pandemic by time (2020 and 2021)

Variables	2020 (Mean, Std. Dev)				2021 (Mean, Std. Dev)			
	LIC	LMIC	UMIC	HIC	LIC	LMIC	UMIC	HIC
New deaths per million people	.06 (.20)	0.44 (2.10)	1.59 (3.18)	1.65 (4.78)	.11 (.39)	.91 (2.26)	3.25 (6.03)	2.57 (7.52)
New cases per million people	2.10 (4.81)	18.01 (47.00)	61.00 (127.45)	89.40 (216.05)	4.75 (14.37)	44.9 (119.94)	158.05 (266.31)	233.50 (483.50)
New tests per thousand	.06 (.08)	.29 (.63)	.53 (.72)	1.99 (2.73)	.14 (.23)	.80 (6.90)	1.48 (1.76)	7.45 (12.48)
Positivity rate	.06 (008)	.09 (.09)	.13 (.13)	.06 (.08)	.09 (.10)	.10 (.10)	.12 (.09)	.06 (.06)
Stringency index	56.56 (22.49)	60.91 (23.17)	65.61 (21.5)	54.93 (20.99)	39.75 (17.49)	50.66 (19.96)	59.34 (15.97)	53.92 (16.26)
Vaccinated people per hundred	–	–	–	–	1.17 (.82)	2.39 (1.28)	2.87 (1.18)	3.42 (16.26)

There were no data in 2020 on percentage of vaccinated people

*LIC* low-income countries, *LMIC* lower-middle-income countries, *UMIC* upper-middle-income countries, *HIC* high-income countries, Vaccination against COVID-19 was first started in December 2020, and vaccine coverage was shown only in 2021

### Fixed effect and lagged model

The daily confirmed incidence was positively correlated with the daily mortality rate in both 2020 and 2021; similarly, the 15-day lag model revealed significant positive correlations. For all income groups except LIC, the daily number of tests was associated with the daily mortality rate; however, in 2021 there was a positive association for all income groups. In 2020, the daily number of tests was associated with the daily mortality rate for all income groups, except for LIC. Meanwhile, in 2021, all groups had a positive association, and the 15-day lag model showed the same results.

In the case of the stringency index, the results were different according to the year and lag. In 2020, there was a positive association in LIC and HIC but a negative association in LMIC. On the other hand, in 2021, there was a positive association between UMIC and HIC. In the 15-day lag model, LIC and HIC were positively correlated and LMIC and UMIC were negatively correlated in 2020. In 2021, all income groups except LIC had a positive association. Vaccinated people per hundred were only analyzed in the year 2021. In both the non-lagged model and the 15-day lag model, there was a positive association in LMIC and a negative association in HIC (Table 2).

**Table 2:** Mortality and incidence rates per million, number of new tests per thousand, positivity rate, stringency index, and vaccinated people per hundred derived from the fixed-effect model by time (2020 and 2021)

	Non-lagged model								15-day lag							
Variables	2020				2021				2020				2021			
	LIC	LMIC	UMIC	HIC	LIC	LMIC	UMIC	HIC	LIC	LMIC	UMIC	HIC	LIC	LMIC	UMIC	HIC
New cases per million	.04^***^(9.6)	.04^***^ (6.5)	.14^***^ (26.5)	.14^***^ (31.5)	.08^***^ (7.2)	.18^***^ (30.3)	.26^***^ (36.4)	.12^**^ (28.3)	.02^***^ (5.6)	.04^***^ (6.6)	.09^***^ (14.1)	.09^***^ (16.6)	.01 (.4)	.01^***^ (0.5)	.04^***^ (3.5)	.05^***^ (10.3)
New tests per thousand	−.16 (−0.4)	.67^***^ (16.6)	.24^***^ (8.4)	.19^***^ (15.2)	.23^**^ (3.0)	.21^***^ (7.3)	.55^***^ (13.7)	.20^***^ (16.4)	.08 (1.9)	.54^***^ (12.4)	.39^***^ (11.3)	.20^***^ (13.3)	.37^***^ (4.9)	.11^***^ (2.5)	.71^***^ (12.9)	.10^***^ (7.3)
Positivity rate	.25^***^ (4.9)	2.31^***^ (28.6)	3.56^***^ (42.3)	5.70^***^ (56.8)	.68^***^ (3.9)	3.57^***^ (32.3)	2.12^***^ (19.5)	6.04^***^ (50.8)	.20^***^ (4.3)	1.69^***^ (19.0)	2.89^***^ (28.9)	4.03^***^ (34.4)	.95^***^ (5.8)	4.27^***^ (29.3)	2.60^***^ (13.4)	4.64^***^ (28.1)
Stringency index	.02^*^ (2.3)	−.06^***^ (−4.0)	.14 (.8)	.54^***^ (28.8)	−.05 (1.3)	−.02 (−2.4)	.49^***^ (10.6)	.53^***^ (27.0)	.02^**^ (3.0)	−.06^***^ (−3.7)	−1.58^***^ (−5.7)	.33^***^ (14.0)	−.17 (−.5)	.12^**^ (1.6)	.66^*^ (2.3)	.88^***^ (37.7)
Vaccinated people per hundred	–	–	–	–	−.01 (−.9)	.03^***^ (4.2)	−.01 (−9.7)	−.15^***^ (− 32.5)	–	–	–	–	.01 (.4)	.05^***^ (6.6)	.10 (1.19)	−.18^***^ (−29.8)
Constant	−.08^**^ (−2.3)	.35^*^ (5.3)	.74^***^ (7.2)	−.69^***^ (−7.9)	−.09^**^ (−2.9)	.32^***^ (6.1)	−2.41^***^ (12.7)	−.1.91^***^ (− 22.62)	−.02 (−.7)	.63^***^ (8.9)	1.88^***^ (14.5)	−.33^***^ (− 3.2)	−.46^*^ (− 3.7)	−.43^***^ (− 2.9)	−2.50^***^ (− 10.2)	−2.60 (− 26.0)
R^2^	.203	.445	.625	.574	.554	.649	.584	.568	.133	.370	0.532	0.442	0.344	0.322	0.459	0.427
Number of countries	9	22	29	44	8	25	31	45	9	21	28	44	8	25	31	45
Observation	1511	4692	6476	11,323	296	2991	4215	11,393	1380	4525	6054	10,856	296	2990	4179	10,829

There were no data in 2020 on percentage of vaccinated people

*LIC* low-income countries, *LMIC* lower-middle-income countries, *UMIC* upper-middle-income countries, *HIC* high-income countries; Vaccination against COVID-19 was first started in December 2020, and vaccine coverage is excluded from the 2020 model; *P < .05, **P < .01, ***P < .0001; F test: Prof > F = 0.0000, Hausman test: Prob > Chi^2^ = 0.0000

### Quadratic regression model

In 2020 and 2021, the slope and direction of COVID-19 pandemic was similar. The daily cases were increased when testing approaches were increased. Were similar overall. There was an overall trend for the daily mortality rate to increase with daily incidence. And when the daily cases exceeded a certain level, the mortality rate increased more rapidly. As daily testing rates increased, in 2020, daily mortality rates also increased in LMIC; however, in 2021, daily mortality rates increased initially and then decreased among all income groups. The stringency index and daily mortality rates and slopes were relatively small. However, in HIC, as the daily mortality decreased, the stringency index also reduced and showed a tendency to increase again after a certain level.

**Fig. 1:**
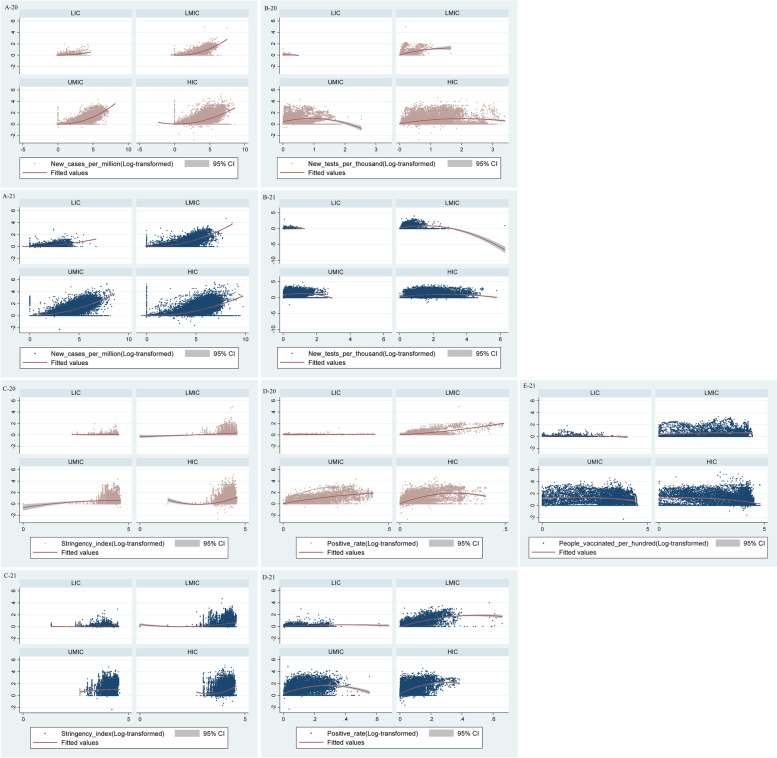
Quadratic lines of the association between **A-** daily mortality and incidence rates per million, **B-** number of new tests per thousand, **C-** stringency index, **D-** positivity rate **E-** people vaccinated per hundred with new deaths per million according to country wealth, estimated by the World Bank. Vaccination against COVID-19 was first started in December 2020, and vaccine coverage was shown only in 2021; ‘20’ is 2020 and ‘21’ is 2021. ex) ‘A- 20’ is the incidence rate in 2020. **A** to **E** Trend of COVID-19 pandemic case distribution, testing, vaccination and stringency index by economic rank of countries by time (2020 and 2021)

In the case of the positivity rate in 2020, LMIC and UMIC increased with mortality, but in HIC, as the positivity rate increased, daily mortality rates tended to increase and then decrease. In 2021, the positivity rate increased with mortality in LMIC, UMIC, and HIC, but decreased when it exceeded a certain level. Lastly, vaccination coverage in 2021 did not appear to change with mortality. However, the mortality rate in HIC did not change initially but decreased when it exceeded a certain level (Fig. 1 in Appendix).

## Discussion

In the present study, the daily mortality and incidence rates, and daily testing rates were all higher in HIC than in other income countries, suggesting that the first year of the COVID-19 pandemic was harsher in the United States, Europe, and other developed countries than elsewhere [42, 43]. These findings explore that COVID-19 has had a greater impact on HIC than on other countries in the first year of the pandemic. Although HIC reported the highest daily testing rate, these countries also reported the lowest positivity rate, likely due to the relatively high number of tests performed [44]. As these countries tested the range of population that was broader than that tested elsewhere, the resulting positivity rate may be lower than that observed elsewhere [45]. It indirectly showed that high GDP per capita is not enough to control public health emergencies like the COVID-19 pandemic because new cases and deaths per million were highest in HIC. High SI means rigid public restrictions and such political decisions may not be perfect for controlling the transmission. It means that smart application of those restrictions considering local context could be more effective in pandemic time.

This is the first study to use the present model; therefore, direct comparisons of findings with previous studies are difficult. Previous studies have reported a positive association between daily incidence and mortality rates, [1] which is consistent with the present study. There was disparity on critical supply, testing of the cases, vaccination [46–48] and structural factors [49] in low-income and middle-income countries and it is in line with our results. The present findings have shown that the daily incidence rate was associated with the daily mortality rate; as the daily incidence rate increased, the increase in the daily mortality rate became steeper. These findings may indicate the challenges associated with infectious disease management that arise when the number of confirmed cases exceeds the capacity of a healthcare system, resulting in patients failing to receive the required care and dying prematurely [50].

Previous studies have suggested that the number of tests performed is negatively associated with mortality rates [51], which is contradictory to the present findings. In a study by Chaudhry [52], the number of tests performed was not associated with the mortality rate. This discrepancy may be due to differences between target countries and the time of data. Another possible explanation for this discrepancy is the between-study differences in study designs. The previous study was cross-sectional, whereas the present study was longitudinal. Finally, a number of previous studies that examined the association between COVID-19 testing and mortality rates account include data from the early days of the pandemic, and thus, exclude data from the third and fourth wave of COVID-19. In contrast, the present study accounted for this period. Previous studies reported that a broad testing strategy resulted in relatively lower case fatality ratio due to the high rate of asymptomatic and mildly symptomatic patients being detected [45], helping to reduce the overall mortality rate [53, 54]. In other words, the number of tests increases the number of asymptomatic patients and patients with mild symptoms can be supported, reducing the mortality rate. However, the association between testing and mortality rates when there is a drastic spike in the number of COVID-19 cases, cannot be explained by this theory. Testing to identify infected individuals is key to preventing the spread of infection [7]. An aggressive testing policy may reduce the associated mortality rate and prevent transmission [53, 55]. The positivity rate was positively associated with the mortality rate in the present study. In addition, the effect value was larger in richer than in poorer countries. In HIC, a relatively high number of tests was performed, resulting in a higher positivity rate than that observed elsewhere, suggesting that more patients were infected with and died of COVID-19 there than elsewhere. In the present study, the positivity rate was associated with increased mortality rates in LIC, LMIC, and UMIC. In contrast, in HIC, when the positivity rate exceeded a certain value, the mortality rate decreased. This finding is difficult to interpret and further research is required to elucidate the interdependence among testing policies, disease severity, and healthcare system capacity.

In all countries, except the LMIC, testing rates increased alongside mortality rates and subsequently decreased. These findings suggest that a high number of tests does not correspond to a high rate of COVID-19 spread. However, an aggressive testing strategy above a certain threshold may help prevent the spread of COVID-19 and enable the delivery of timely interventions to affected patients, thus reducing the mortality rate.

The stringency index is an indicator of the extent of government control measures, whereby higher values represent greater limitations on population mobility and person-to-person contacts. However, this index is not necessarily positively associated with the incidence, mortality, or fatality rates. In our study, SI is positively associated with HIC but negatively associated with UMIC and LMIC. The results are partially similar with the research by Oghenowede Eyawo, A. M. Viens & Uchechukwu Chidiebere Ugoji in 2021 [56] and Morgan Pincombe, Victoria Reese, Carrie B Dolan 2021 [57]. A previous cross-sectional study has shown no association between the stringency index and the case fatality ratio [55, 58]. Moreover, previous studies have shown that lockdown was not associated with mortality rates [52]. However, this does not necessarily mean that government-imposed restrictions have no effect on COVID-19 spread. In fact, Chaudhry [52] has shown that lockdowns may reduce daily incidence rates despite having no direct impact on mortality rates. In contrast, a study by Sorci reported that countries that implement severe restrictions experienced higher mortality rates; this finding may be due to the fact that countries less affected by this pandemic may have implemented milder restrictions [59]. While the strictness of policy may prevent disease spread and reduce mortality rates, the reverse association may also exist, whereby high mortality rates result in stricter policy, suggesting an overall positive and bidirectional association between the two variables [60].

In the present study, the stringency index and mortality rates were positively associated in LIC and HIC, indicating that countries with higher mortality rates may have implemented stricter policies. High mortality rates have triggered policies such as travel restrictions and lockdowns. Among the four groups of countries, HIC had the lowest stringency index, while it had the highest mortality and incidence rates. In fact, an increase in the stringency index resulted in a steeper increase in mortality rates in HIC, suggesting that control measures were strengthened as mortality rates increased, and that several types of control measures were implemented concurrently once the mortality rates exceeded a certain threshold. Meanwhile, in LMIC and UMIC, mortality rates were negatively associated with the stringency index, suggesting that a low level of restrictions was present despite high mortality rates, while a high level of restrictions may have reduced mortality rates in the early stages of the pandemic. To date, there have been no studies on the relationship between the stringency index and daily mortality rates associated with COVID-19 in LMIC and UMIC. LIC, LMIC, and UMIC had low coefficients, and it was difficult to determine that LMIC and UMIC showed a negative association in the fitted line. Further studies are required to elucidate the relationships between these variables in these countries. In the period of our study, the worldwide vaccination rate is still extremely low, and this is the result excluding the vaccine effect. Therefore, rather than the period after vaccination in this study, the emergence of a new strong mutant virus of SARS-CoV2 or the occurrence of a new infectious disease can give greater implications in the period when there is no vaccine.

## Conclusion

There is no consistent trend in case findings, vaccination, and public restriction policies (mentioned by the stringency index here) during COVID-19 pandemic. More resource, technology and restriction policies should be applied considering the magnitude of cases, severity and high risk areas of transmission other than the economic rank of countries. More research collaboration and replication of effective policies are greatly important.

## Limitations and future research

The present study has several strengths. First, this was a longitudinal study that contributed evidence that was not possible to obtain in previous cross-sectional studies. As the present study included data from > 100 countries, the present findings are likely globally generalizable but carefully. Second, to the best of our knowledge, the present study was the one of the earliest to identify the associations among COVID-19-related mortality and testing rates, positivity rate, and the stringency index at the global level. Based on our results, an application of these findings could be a formulation of policies to control the pandemic considering multiple factors like the GDP per capita, supply chain trends of logistics, individual and family protection protocols and supply of COVID-19 vaccines based on needs. Now, the pandemic seems to be resolved but it needs regular research and surveillance worldwide because available data are not sufficient to cope with the situation for the next stage or future pandemic. Moreover, it may help to enhance the health delivery system to cope possible future pandemic observing 2 year COVID-19 trend.

The present study has some limitations. One of the important limitations is that it is inherently difficult to compare countries globally given that there are significant contextual variations in which policies are implemented. Take-up rates of these policies may also affect the effectiveness of these policies. Similarly, this longitudinal study performed analyses with a day as a time unit. Consequently, population age and density, which may affect mortality rate estimates, were not considered in the present study. Second, the present study used data acquired from several sources. The most important limitation of this study is that the political system, enabler or a barrier in taken population level decisions and how these decisions are followed up. The timing of decisions taken for public restriction, it’s monitoring and strictly follow up influence the outcome of COVID-19 trend. Finally, different stages of the pandemic may have followed distinct patterns; in particular, our findings are data from a period when vaccines are not yet widely distributed worldwide, and vaccine effects are not considered.

## Data Availability

The data are available from Our World in Data. If you need the processed data, please contact the author to request the data.

## Appendix

Table 3

**Table 3:** Variance Inflation Factor (VIF) and tolerance of each variable by time (2020 and 2021)

Variables	2020		2021	
	Variance Inflation Factor	Tolerance (1/VIF)	Variance Inflation Factor	Tolerance (1/VIF)
New cases per million	3.17	0.31	2.12	0.47
New tests per thousand	2.39	0.42	2.14	0.46
Positivity rate	2.12	0.47	1.88	0.53
Stringency index	1.10	0.91	1.14	0.87
Vaccinated people per hundred	–	–	1.28	0.78
Mean	2.20		1.71	

Vaccination against COVID-19 was first started in December 2020, and vaccine coverage was shown only in 2021

Fig. 1

## Abbreviations

FEM: Fixed effect model
GNI: Gross national income
HIC: Higher income countries
LIC: Lower income countries
LMIC: Lower-middle income countries
MERS-CoV: Middle East respiratory syndrome
OLS: Ordinary least square
SARS-CoV: Severe acute respiratory syndrome
UMIC: Upper-middle income countries
WHO: World Health Organization
